# HIV infection of the male genital tract – consequences for sexual transmission and reproduction

**DOI:** 10.1111/j.1365-2605.2009.00973.x

**Published:** 2010-02

**Authors:** A Le Tortorec, N Dejucq-Rainsford

**Affiliations:** INSERM U625, Rennes; Rennes I University, Groupe d’Etude de la Reproduction chez l’Homme et les MammifèresIFR 140, Campus de Beaulieu, Rennes, France

**Keywords:** assisted reproductive techniques, epididymis, germ cells, highly active antiretroviral therapy, human immunodeficiency virus, male genital tract, prostate, reservoir, semen, seminal vesicle, spermatozoa, testis

## Abstract

Despite semen being the main vector of human immunodeficiency virus (HIV) dissemination worldwide, the origin of the virus in this bodily fluid remains unclear. It was recently shown that several organs of the male genital tract (MGT) are infected by HIV/simian immunodeficiency virus (SIV) and likely to contribute to semen viral load during the primary and chronic stages of the infection. These findings are important in helping answer the following questions: (i) does the MGT constitute a viral reservoir responsible for the persistence of virus release into the semen of a subset of HIV-infected men under antiretroviral therapy, who otherwise show an undetectable blood viral load? (ii) What is the aetiology of the semen abnormalities observed in asymptomatic HIV-infected men? (iii) What is the exact nature of the interactions between the spermatozoa, their testicular progenitors and HIV, an important issue in the context of assisted reproductive techniques proposed for HIV-seropositive (HIV+) men? Answers to these questions are crucial for the design of new therapeutic strategies aimed at eradicating the virus from the genital tract of HIV+ men – thus reducing its sexual transmission – and for improving the care of serodiscordant couples wishing to have children. This review summarizes the most recent literature on HIV infection of the male genital tract, discusses the above issues in light of the latest findings and highlights future directions of research.

## Introduction

Shortly after the first cases of acquired immuno-deficiency syndrome (AIDS) were described in 1981 in the United-States, two main populations at risk were identified, homosexual men and haemophiliacs. Based on these observations, the cause of AIDS was hypothesized to be due to a sexually and blood transmitted pathogenic agent, even before the discovery of its aetiological agent, the human immunodeficiency virus (HIV). Twenty six years later, the spread of HIV has become a global phenomenon and infected more than 65 million people of whom 25 million are deceased, according to the latest estimates. In 2007, 2.7 million people were newly infected, 80% of whom were through sexual transmission.

Semen represents the main vector of HIV dissemination, evidenced by transmission occurring more efficiently from men to women and men than from women to men (Royce *et al.*, 1997). The origin of the free viral particles and infected cells contaminating semen are still unclear. Semen is composed of cells and secretions from the testes, epididymis, prostate, seminal vesicles and bulbo-urethral glands. Because of the extreme difficulty in sampling the genital organs from asymptomatic HIV+ men, the infection of the semen-producing organs has been little studied and several questions remain:

1. What is the source of virus production in the male genital tract (MGT)? It is established that the viral strains present in semen do not solely arise from the blood compartment but is/are the MGT organ(s) responsible for the seminal viral load?
2. What is the nature of the interactions among HIV, the spermatozoa and their progenitor cells the testicular germ cells?
3. Does the MGT constitute a viral reservoir resistant to current anti-HIV therapy? Highly Active Antiretroviral Therapy (HAART) does not always eradicate the virus from semen, even when achieving an undetectable viral load in blood and can favour the sexual transmission of drug-resistant strains;
4. What is the cause of the semen abnormalities recently described in HIV+ men?

Answering these questions is crucial for the design of new therapeutic strategies aimed at eradicating the virus from the genital tract of HIV+ men (thus reducing its sexual transmission) and for improving the care of serodiscordant couples wishing to have children. The aim of this review was to summarize the current knowledge on HIV infection of the male genital tract, discuss the above questions in view of the latest findings and highlight future directions of research.

## Origin of HIV in semen?

HIV is present in semen as free viral particles and infected cells. It was originally believed that the only source of HIV in semen was infected lymphocytes and macrophages coming from the blood. It has now been shown that the HIV strains present in semen evolve separately from the strains in the blood or in other anatomical compartments (Kroodsma *et al.*, 1994; Vernazza *et al.*, 1994; Zhu *et al.*, 1996; Byrn *et al.*, 1997; Coombs *et al.*, 1998; Eron *et al.*, 1998; Hecht *et al.*, 1998; Kiessling *et al.*, 1998; Eyre *et al.*, 2000; Gupta *et al.*, 2000; Ping *et al.*, 2000; Ghosn *et al*., 2004b;Pillai *et al.*, 2005). This indicates that the MGT constitutes a viral compartment distinct from the blood and locally produces viral particles that are under a specific selective pressure. Phylogenetic analyses showed that the free viral particles contaminating the seminal fluid are, in some cases, distinct from those isolated from the infected leucocytes present in semen (Paranjpe *et al.*, 2002; Ghosn *et al*., 2004b), while a subset arise because of passive diffusion from the blood (Curran & Ball, 2002; Ghosn *et al*., 2004b). In addition, a discrepancy between the number of infected leucocytes in semen and the viral load in the seminal plasma is often observed [reviewed in (Dejucq & Jegou, 2001; Dejucq-Rainsford & Jegou, 2004)]. Thus it appears that the seminal lymphocytes and macrophages are not the only producers of the viral particles detected in the seminal fluid and that a distinct productive source contributes to virus shedding in semen. Of interest is that HIV shedding in semen may be intermittent, a phenomenon yet to be explained and not linked to variations in the blood viral load (Coombs *et al.*, 1998; Gupta *et al.*, 2000; Bujan *et al.*, 2004).

As infected leucocytes in semen produce viral strains that are different from those in blood leucocytes (Kroodsma *et al.*, 1994; Vernazza *et al.*, 1994; Zhu *et al.*, 1996; Byrn *et al.*, 1997; Coombs *et al.*, 1998; Eron *et al.*, 1998; Hecht *et al.*, 1998; Kiessling *et al.*, 1998; Eyre *et al.*, 2000; Gupta *et al.*, 2000; Ping *et al.*, 2000; Ghosn *et al.*, 2004a, 2004b; Pillai *et al.*, 2005), this indicates that the infected leucocytes and the free virions contaminating semen have distinct origins within the male genital tract, therefore suggesting that several semen-producing organs are infected and contribute either free virus or infected cells. The potential sources of virus in the MGT are discussed below.

## HIV detection within the male genital tract organs and cells

### HIV & spermatozoa

The nature of HIV interaction with spermatozoa is still a matter of debate (Piomboni & Baccetti, 2000). Although the main HIV receptor CD4 is absent from spermatozoa, several alternative receptors for HIV have been described: GalAAG, a glycolipid related to galactosylceramide (an alternative receptor used by HIV in conjunction with a chemokine co-receptor to enter CD4-negative cells) (Clapham *et al.*, 1999), is expressed by about 30% of ejaculated spermatozoa and binds the HIV envelope protein gp120 (Brogi *et al.*, 1998; Gadella *et al.*, 1998). However, this binding is inhibited by seminal plasma (Gadella *et al.*, 1998), suggesting that HIV could not bind to ejaculated spermatozoa through GalAAG, although this alternative receptor could allow HIV binding to testicular or epididymal spermatozoa. The HIV co-receptor CCR5 is also expressed by spermatozoa and could, in principle, allow the fusion of HIV with spermatozoa if present in association with GalAAG (Muciaccia *et al*., 2005a, 2005b). The proteins and/or mRNAs of several other chemokine receptors are expressed by spermatozoa (Isobe *et al.*, 2002; Zhang *et al.*, 2004), some of them previously described to mediate HIV binding. Another receptor allowing the CD4-independent binding and entry of HIV in other cell types (Liu *et al.*, 2004), the mannose receptor, was identified in about 10% of ejaculated spermatozoa and shown to bind HIV gp120 (Fanibunda *et al.*, 2008). No internalization of gp120 was observed and it is presently unknown whether this receptor can trigger the internalization of a whole virion (Fanibunda *et al.*, 2008). In agreement with the finding that spermatozoa express at their surface several molecules able to bind HIV particles, purified motile spermatozoa from HIV negative donors incubated with HIV in the absence of seminal plasma were shown to act as carriers and transmit the virus to susceptible leucocytes in vitro (Dussaix *et al.*, 1993). Using electron microscopy, different groups visualized HIV particles and immuno-labelled proteins attached or internalized into purified spermatozoa from HIV negative donors exposed to HIV *in vitro* (Bagasra *et al.*, 1988; Dussaix *et al.*, 1993; Baccetti *et al.*, 1994; Scofield *et al.*, 1994). One of them reported the detection of HIV particles in the ejaculated spermatozoa of HIV-positive men (Baccetti *et al.*, 1994) and their transfer to oocytes in fertilization experiments in vitro (Baccetti *et al.*, 1994) but these results have not been confirmed (Pudney *et al.*, 1999).

It is generally accepted that motile spermatozoa are not productively infected by the virus. Although HIV DNA and/or RNA were detected by polymerase chain reaction (PCR) in spermatozoa purified using a gradient of Percoll without swim-up (which further separates motile spermatozoa from non-motile), these positive finding were assumed to result from contaminations of this fraction by a few remaining infected leucocytes or from false positives (Dulioust *et al.*, 1998; Marina *et al.*, 1998; Tachet *et al.*, 1999; Hanabusa *et al.*, 2000; Leruez-Ville *et al*., 2002a). Indeed when PCR is performed on the highly purified motile spermatozoa fraction isolated from semen through a percoll gradient + swim-up or double tube techniques, HIV nucleic acids are generally not detected (Quayle *et al.*, 1997; Kim *et al.*, 1999; Hanabusa *et al.*, 2000; Pasquier *et al.*, 2000; Bujan *et al.*, 2004; Politch *et al.*, 2004; Kato *et al.*, 2006; Persico *et al.*, 2006; Bostan *et al.*, 2008). Of note, however, positive results were sometimes obtained even on this highly purified spermatozoa fraction (Baccetti *et al.*, 1994; Chrystie *et al.*, 1998), which could result from the persistence of free viral particles despite the washes in the presence of high seminal viral load (Fiore *et al.*, 2005). The early report of HIV DNA within both motile and non-motile spermatozoa using in situ PCR (Bagasra *et al.*, 1994) could not be confirmed by two other teams (Pudney *et al.*, 1999; Persico *et al.*, 2006). In summary, most studies failed to detect HIV nucleic acids within purified motile spermatozoa, indicating that the few positive results reported represents either false positives or very rare events. Of note is that only a very small fraction of the millions of ejaculated spermatozoa are tested in PCR, inducing sampling biases which may also account for the divergent results amongst studies. Interestingly, however, a recent study using a range of techniques demonstrated the presence of HIV DNA in a subset of ejaculated spermatozoa with an abnormal morphology (Muciaccia *et al.*, 2007) – a cell population that had not been studied so far apart from the two early studies, indicating HIV interaction with non-motile spermatozoa using electron microscopy (Scofield *et al.*, 1994) or in situ PCR (Bagasra *et al.*, 1994). A phenomenon to bear in mind in the context of HIV detection within spermatozoa is the fact that mammalian spermatozoa spontaneously take up foreign DNA or RNA in the absence of seminal plasma (e.g. epididymal spermatozoa). Spermatozoa have the ability to reverse-transcribe RNA of viral origin into cDNA fragments, internalize them into sperm nuclei (and even in some instances integrate the foreign DNA into the sperm genome) and transfer the foreign nucleic acids to embryos upon in vitro fertilization (Spadafora, 1998; Giordano *et al.*, 2000). Sperm interaction with foreign nucleic acids molecules triggers endogenous nucleases that cleave both exogenous and genomic DNA, eventually leading to cell death (Spadafora, 1998). In the context of HIV infection, one hypothesis is that non-specific uptake of HIV RNA/DNA by epididymal spermatozoa would lead to abnormal spermatozoa prone to cell death, which may explain the detection of ejaculated HIV DNA+ abnormal spermatozoa by Muciaccia *et al.* (Muciaccia *et al.*, 2007). Alternatively, this detection may result from specific interactions between HIV and spermatozoa.

In conclusion, spermatozoa display several receptors that could allow HIV specific binding during their progression through the male genital tract. Thus it is likely that spermatozoa can act as a carrier for viral particles encountered within the testis or epididymis, i.e. in the absence of seminal plasma, an inhibitor for some of the HIV receptors present on spermatozoa. It is established that spermatozoa do not produce HIV particles. Whether they can support the early steps of HIV replication (e.g. up to viral DNA synthesis) as proposed by some authors remains speculative, as the vast majority of studies did not evidence any HIV genetic material and spermatozoa are considered as metabolically inert cells. However, non-specific mechanisms such as foreign RNA uptake could be at play and explain the detection of HIV DNA in a subset of abnormal spermatozoa. The exact nature of the interactions between HIV and spermatozoa, and their impact on spermatozoa morphology, are far from fully understood and require further studies.

### HIV & the testis

The infection of the testis by HIV can have important consequences for the eradication of the virus from the MGT by antiretroviral therapies. Thus, the existence of the blood testis barrier and of the drug efflux pumps of the ABC transporter family expressed by a wide range of testicular cell types, restrict the drug access to this organ, as shown for some HIV replication inhibitors (Choo *et al.*, 2000; Livni *et al.*, 2004). These data indicate that, if infected, the testis may represent a viral sanctuary resistant to antiviral treatments. To date, the concentration of HIV inhibitors in the human testis and in other organs of the MGT is unknown. To investigate the susceptibility of the human testis to HIV infection, our team developed an organotypic culture of this organ (Roulet *et al*., 2006a) and revealed that the human testis is infected by HIV-1 ex vivo and produces low levels of infectious viral particles (Roulet *et al.,* 2006b). The main virus-producing cells in this culture model are the resident testicular macrophages (Roulet *et al.,* 2006b).The analysis of the in vivo infection of the testis in asymptomatic macaques infected by SIV, the simian counterpart of HIV, was recently undertaken. The experimental infection of cynomolgus macaques represents the best animal model to study HIV infection as the animals display many common features with HIV infected humans throughout the disease progression (such as the nature of the infected organs/cells and the immune responses) and they develop AIDS like humans (Haigwood, 2004). The in vivo study of SIV-infected macaques confirmed the productive infection of the testis during the asymptomatic chronic stage and revealed that this infection occurs during the acute primary infection (Le Tortorec *et al*., 2008a). Infected cells within the testicular interstitial tissue are macrophages and T lymphocytes, a finding in agreement with observations in chronically-infected juvenile pigtail macaques (Shehu-Xhilaga *et al.*, 2007) and in asymptomatic HIV+ men (Muciaccia *et al.*, 1998; Paranjpe *et al.*, 2002). Importantly, the presence of SIV in some isolated testicular germ cells was shown for all the animals tested, using both immunohistochemistry and in situ hybridization (Le Tortorec *et al.*, 2008a), thus reinforcing the previous controversial observations in macaques and humans using other techniques (Muciaccia *et al.*, 1998; Shehu-Xhilaga *et al.*, 2007). The spermatogenesis and testicular morphology in chronically-infected macaques and humans appeared normal (Muciaccia *et al.*, 1998; Le Tortorec *et al*., 2008a). A transitory increase in the plasmatic testostestone level of chronically infected macaques was observed, without any significant modifications of the Luteinizing Hormone (LH) level (Le Tortorec *et al*., 2008a). In humans, an increase in testosterone level was similarly reported in some chronically-infected patients (Christeff *et al.*, 1992). In patients with AIDS, a decrease in circulating androgens is frequently encountered (Lo & Schambelan, 2001), which can occur in the presence of normal or elevated LH level, indicating a primary testicular failure. To test the hypothesis of a direct effect of the virus on the steroidogenic function of Leydig cells, the susceptibility of this cell type to HIV infection in vitro was examined using a range of HIV and SIV strains with various cell tropisms. The results demonstrated that human Leydig cells are susceptible to some specific HIV-2 and SIV strains but are not infected by HIV-1 strains (Willey *et al.*, 2003; Roulet *et al.*, 2006b). Thus the testosterone level modifications in HIV-1 infected individuals are unlikely to be caused by a direct effect of the virus on Leydig cells but more probably result from the altered hypothalamo-pituitary axis or, in cases where normal pituitary hormones levels are observed, from modified cytokines production within the testis or from direct interactions between Leydig cells and infected macrophages (Le Tortorec *et al.,* 2008a).

During the later stages of the disease, the testis morphology is severely damaged (Dejucq & Jegou, 2001), with different levels of testicular germ cell degeneration leading in some cases to a Sertoli cell only-syndrome. This most probably results from the decrease in testosterone level, elevated body temperature and presence of opportunistic infections rather than from the germ cell infection described in some studies (Da Silva *et al.*, 1990; Nuovo *et al.*, 1994; Muciaccia *et al.*, 1998), as normal testicular morphology is observed during the asymptomatic stage despite the association of HIV/SIV with testicular germ cells.

To conclude, recent data show that the testis is infected early during the course of HIV infection. This infection is not associated with either any apparent change in testicular morphology or inflammation of the organ (Le Tortorec *et al.*, 2008a). Testicular leucocytes represent the main target cells for the virus, but testicular germ cells also occasionally associate with HIV. This is important to bear in mind for the practice of intra cytoplasmic sperm injection (ICSI) using testicular germ cells from HIV-infected individuals. The presence of HIV receptors on human testicular germ cells and the molecular interactions between these cells and HIV is currently being investigated. Whether the testis constitutes a viral sanctuary despite antiretroviral therapy is under study.

### HIV & the accessory glands

Early studies showed evidence of HIV and SIV in immune cells infiltrating the epididymis, prostate and seminal vesicles of men (Da Silva *et al.*, 1990; Pudney & Anderson, 1991; Nuovo *et al.*, 1994) or macaques respectively (Miller *et al.*, 1994) at the AIDS stage. But what happens during the early and asymptomatic stages of the disease has not been studied.

We recently analysed the infection of the epididymis, prostate and seminal vesicles in primary- and chronically-infected macaques. All these MGT organs are infected very early and produce viral particles. The infection persists during the chronic stage and its intensity is positively correlated with the blood viral load. Infected cells are mainly T lymphocytes and to a lesser extent, macrophages. The presence of infected leucocytes during the chronic stage was similarly reported in the epididymis of SIV-infected pigtail macaques (Shehu-Xhilaga *et al.*, 2007). The infected immune cells are mainly localized within the stroma of the organs but are also found inserted within the epithelium, a finding most common within the epididymis. Their localization within the secretory epithelium could lead to the release of free viral particles and infected cells in the lumen and therefore in the seminal plasma during ejaculation. In addition, the viral particles produced within the stroma may be sequestrated by the epithelial cells – a phenomenon described for prostatic cells in vitro (Dezzutti *et al.*, 2001) – and subsequently released in the seminal fluid. The chronic infection of the accessory glands is associated with T-cell infiltrations and production of inflammatory cytokines. The prostate and seminal vesicles systematically displayed higher levels of infection than the epididymis and the testis. As prostate and seminal vesicles secretions account for respectively 30% and 60% of the seminal fluid (Wolff, 1995), these two organs are likely to represent the main source of virus in semen. In favour of this hypothesis, prostatic massage in men was shown to significantly increase the seminal viral load (Smith *et al.*, 2004). In the same way, the previous studies indicated that vasectomy had little effect on the semen viral load (Anderson *et al.*, 1991; Krieger *et al.*, 1998), suggesting that the testis and epididymis are minor HIV contributors in semen. We recently confirmed that the human prostate is susceptible to HIV-1 infection *ex vivo* (Le Tortorec *et al.*, 2008b). Interestingly, the prostate was preferentially infected by HIV-1 R5 strains (the sexually-transmitted strains) compared with X4 strains, which are not sexually transmitted and appear during the later stages of the disease (Le Tortorec *et al*., 2008b).

## The male genital tract – a viral reservoir?

HAART aims for durable suppression of viral load, restoration and/or preservation of immunological function and has dramatically enhanced the quality and life span of the individuals who benefit from these treatment. However, the existence of viral sanctuaries prevents the eradication of the virus in the body. A viral sanctuary is either an anatomical (e.g. the brain) or a cellular (e.g. the latently infected resting memory lymphocytes) site, impermeable to the action of one or several antiviral drugs and within which the virus replicates or persists despite treatment. Such sanctuaries are called reservoirs when they replenish the body in free virus or infected cells. Thus when HAART is discontinued, the blood plasma viral load that was undetectable under the pressure of the antiretroviral drugs systematically rises again from these reservoirs (Davey *et al.*, 1999).

In semen, some viral inhibitors display sub-optimal concentrations (Kashuba *et al.*, 1999; Taylor & Pereira, 2001; Lafeuillade *et al.*, 2002; Ghosn *et al*., 2004a; Chan *et al.*, 2008), sometimes leading to the emergence of drug-resistant strains (Eron *et al.*, 1998;Eyre *et al.*, 2000; Taylor *et al.*, 2001, 2003; Ghosn *et al*., 2004a, 2004b) and to their sexual transmission (Hecht *et al.*, 1998; Grant *et al.*, 2002; Little *et al.*, 2002; Markowitz *et al.*, 2005). The testis is a well-known pharmacological sanctuary into which HIV inhibitors have restricted access (Choo *et al.*, 2000; Livni *et al.*, 2004). The organ is thus a prime candidate for an HIV sanctuary. The fact that the rate, the kinetic of emergence and the diversity of drug-resistant strains diverge between the blood and the seminal plasma (Kroodsma *et al.*, 1994; Eron *et al.*, 1998; Eyre *et al.*, 2000; Ghosn *et al.*, 2004b; La Sala *et al.*, 2007) strongly suggest that HIV in semen arises from a biological compartment separate from blood.

Several studies indicate that the MGT may constitute a viral reservoir responsible for HIV shedding in semen [reviewed in (Dejucq & Jegou, 2001; Dejucq-Rainsford & Jegou, 2004)]. Although in most patients HAART can reduce the semen viral load to an undetectable level (Liuzzi *et al.*, 1999; Barroso *et al.*, 2000; Vernazza *et al.*, 2000; Leruez-Ville *et al.*, 2002b; Bujan *et al.*, 2004; Chan *et al.*, 2008; Ghosn *et al.*, 2008), the persistence of HIV RNA and/or infected cells in semen has been reported in up to 10% of men under various antiretroviral treatment combinations, despite an undetectable blood viral load (Kiessling *et al.*, 1998; Zhang *et al.*, 1998; Dornadula *et al.*, 1999; Mayer *et al.*, 1999; Lafeuillade *et al.*, 2002; Leruez-Ville *et al.*, 2002b; Solas *et al.*, 2003; Vernazza *et al.*, 2007; Marcelin *et al.*, 2008). Most recently, an even higher percentage of men shedding HIV in semen despite effective HAART has been reported: 4 months following the initiation of HAART, 48% of 25 individuals with suppressed blood viral load were releasing HIV in semen and for 16% of them at a high level. Strikingly, this persistent shedding was still observed for 31% of 13 individuals under effective HAART for a median of 10 years (Sheth *et al.*, 2009). The residual viral load detected in semen is generally low but appears to be extremely variable amongst individuals (range of 0.6 log to 5 log of HIV RNA copies/mL) (Dornadula *et al.*, 1999; Mayer *et al.*, 1999; Vernazza *et al.*, 2000; Leruez-Ville *et al*., 2002b; Bujan *et al.*, 2004; Marcelin *et al.*, 2008; Pasquier *et al.*, 2008).

This persistence of HIV excretion in the semen of a subset of individuals under HAART has potentially important consequences for the transmission of the virus. The critical inoculum for sexual transmission is unknown. A mathematical model indicated a very low risk of heterosexual transmission of three of 10 000 episodes of intercourse for 3 Log of HIV RNA copies per ejaculate (Chakraborty *et al.*, 2001) and HIV heterosexual transmission was estimated to decrease by about 80% following HAART (Castilla *et al.*, 2005). Thus the question has been raised as to whether natural conception is an acceptable/negligible risk option for HIV-serodiscordant couples in whom the HIV+ partner is effectively treated (Barreiro *et al.*, 2007) and recently, the Swiss national AIDS Commission stated that serodiscordant couples in whom the infected partner had an undetectable blood viral load under prolonged HAART could safely have unprotected intercourses in the absence of other sexually transmitted infections (STIs) (Vernazza *et al.*, 2008) – a factor known to increase HIV shedding in semen (Dejucq-Rainsford & Jegou, 2004; Zuckerman *et al.*, 2009). This statement has generated a heated debate amongst scientists and patients’ associations. A mathematical model calculated that HIV incidence would quadruple if this advice was followed by 10 000 couples over 10 years (Wilson *et al.*, 2008). It appears from the literature that although drastically reduced, the possibility of transmitting HIV despite an undetectable blood viral load following HAART remains in a subset of patients, as recently illustrated by the case report of an homosexual man under effective HAART who contaminated his partner (Sturmer *et al.*, 2008).

In conclusion, the fact that HIV may persist and shed intermittently in semen from men under effective HAART free of other STIs (Bujan *et al.*, 2004; Marcelin *et al.*, 2008) reinforces the need for testing not only the blood viraemia but also the seminal viral load and this in several distant measurements, to assess the level of risk of a sexual transmission of each individual under HAART. It also stresses the importance of determining the nature of the reservoirs responsible for HIV shedding in semen, to design more effective therapeutic strategies that can eradicate the virus from semen.

## HIV and male reproduction

In 2008, the majority of the 33 million HIV-infected people worldwide were in their reproductive years. The HIV seropositive male partner of a growing number of serodiscordant couples is seeking assisted reproductive techniques (ART) to have children without contaminating the partner and embryo. ART uses spermatozoa isolated from the infected components in semen (infected immune cells and seminal fluid) and tested negative for viral DNA/RNA (reviewed in (Englert *et al.*, 2004; Bujan *et al*., 2007a)). Semen quality is an essential criterion for inclusion (as an important number of spermatozoa have to be tested) and this represents a limiting factor for many couples. The semen parameters of HIV+ men have been analysed by several teams. In all studies except one (Garrido *et al.*, 2005), a trend towards semen degradation was observed in men at the advanced stage of the disease with low CD4 + T lymphocytes number in blood (Krieger *et al*., 1991a, 1991b;Crittenden *et al.*, 1992; Politch *et al.*, 1994; Dondero *et al.*, 1996; Lasheeb *et al.*, 1997; Muller *et al.*, 1998). As for asymptomatic HIV+ men in the chronic stage, most studies, apart from two (Krieger *et al*., 1991a, 1991b; Garrido *et al.*, 2005), reported various abnormalities: spermatozoa reduced motility, decreased total number and/or increased morphological abnormal forms (Crittenden *et al.*, 1992; Dondero *et al.*, 1996; Muller *et al.*, 1998; Dulioust *et al.*, 2002; Nicopoullos *et al.*, 2004; Bujan *et al*., 2007b; La Sala *et al.*, 2007), lower volume of the ejaculate (Muller *et al.*, 1998; Dulioust *et al.*, 2002; Nicopoullos *et al.*, 2004; Bujan *et al.*, 2007b; La Sala *et al.*, 2007), increased pH of the ejaculate (Bujan *et al.*, 2007b), increased number of round cells in the seminal fluid (Crittenden *et al.*, 1992; Dulioust *et al.*, 2002). The most common findings in recent studies were a decrease in spermatozoa motility and ejaculate volume in healthy HIV+ men (Dulioust *et al.*, 2002; Nicopoullos *et al.*, 2004; Bujan *et al.*, 2007b). An elevated rate of spontaneous abortions was reported in serodiscordant couples of whom the man is HIV+ (Sergerie *et al.*, 2004). Of note is that the majority of these men are taking antiretroviral drugs which may affect semen quality, independently or in addition to the infection. The nucleosidic inhibitors of reverse transcriptase (NRTI), in particular thymidine analogues, are suspected to generate modifications of mitochondrial DNA, which could affect spermatozoa motility (Sergerie *et al.*, 2004; La Sala *et al.*, 2007). Recently, a study examined the semen parameters of HIV+ men before and after the initiation of different combination of antiretroviral therapies (van Leeuwen *et al*., 2008b). A decrease in motile spermatozoa was observed as early as 4 weeks post-treatment initiation, while the semen quality was stable over 1.5 years in untreated HIV+ men (van Leeuwen *et al*., 2008b), suggesting a direct effect of the antiretroviral drugs on spermatozoa motility. Surprisingly, this effect was observed to be independent of the use of thymidine analogues (van Leeuwen *et al.*, 2008b). – Importantly, the semen volume was unchanged following treatment but was in the lower normal range (van Leeuwen *et al*., 2008a, 2008b). Further studies will be crucial to the elucidation of the mechanisms responsible for the semen parameter deterioration observed in healthy asymptomatic HIV+ men.

## Conclusions

Deciphering the origins of HIV in semen is crucial to the development of targeted therapeutic strategies aimed at eradicating the virus in semen. It was recently revealed that MGT organs are infected by HIV during the acute and asymptomatic chronic stages of HIV infection. These organs most likely contribute an important proportion of the semen viral load.

Future research should be directed to the following areas: (i) determining whether one or several of the infected MGT organs constitute a viral reservoir, which could explain the persistence of HIV in the semen of men under effective treatment with undetectable blood viral load; (ii) determining the aetiology of the semen parameter modifications in HIV+ men under HAART; (iii) deciphering the exact nature of the interactions between HIV, the testicular germ cells and the spermatozoa, both important issues in the context of ART; (iv) analysing the effect of HIV infection on the seminal plasma composition and its impact on HIV infectivity, which may reveal new mechanisms that could be useful in the fight against the AIDS pandemic as a few studies suggest that seminal plasma factors may influence HIV sexual transmission.
